# Asymptomatic Pulmonary Artery Intimal Sarcoma with Chest Wall Metastasis as an Initial Manifestation: An Autopsy Case

**DOI:** 10.1155/2018/6153658

**Published:** 2018-05-27

**Authors:** Tomoo Kakimoto, Mamoru Sasaki, Shojiroh Morinaga, Robert Nakayama, Naoto Minematsu

**Affiliations:** ^1^Department of Medicine, Hino Municipal Hospital, 4-3-1 Tamadaira, Hino-shi, Tokyo, Japan; ^2^Department of Diagnostic Pathology, Hino Municipal Hospital, 4-3-1 Tamadaira, Hino-shi, Tokyo, Japan; ^3^Department of Orthopaedic Surgery, School of Medicine, Keio University, 35 Shinanomachi, Shinjuku-ku, Tokyo, Japan

## Abstract

Pulmonary artery intimal sarcoma (PAIS) is a rare mesenchymal malignancy arising in the pulmonary trunk or proximal pulmonary artery and shows intraluminal growth. Clinical manifestations in PAIS are predominantly related to the pulmonary artery embolism, so cases with initial symptoms related to an extrapulmonary metastasis are unusual. The present report describes an 82-year-old man without any cardiopulmonary symptoms who was detected with an abnormal shadow on chest radiography during a routine health checkup. Contrast medium-enhanced chest computed tomography revealed an enhancing mass in the right pulmonary artery, pulmonary nodules, and a chest wall tumor corresponding to the abnormal shadow observed using chest radiography. A core needle biopsy for the chest wall tumor determined a pathological diagnosis of unclassified sarcoma. The patient was diagnosed with PAIS on the basis of clinical, radiological, and pathological correlations. He was scheduled to receive supportive care, but died of respiratory failure 1 year from the first visit. An autopsy revealed the pleomorphic sarcoma occupying the entire lumen of the right pulmonary artery with the only site of extrapulmonary metastasis in the chest wall. We should be aware of rare cases of asymptomatic PAIS found through routine health checkups.

## 1. Introduction

Intimal sarcoma is a rare mesenchymal malignancy arising in the intimal layer of the aorta or pulmonary artery and predominantly shows intraluminal growth [1]. The incidence of pulmonary artery intimal sarcoma (PAIS) is twice as more common than that of aortic intimal sarcoma, while the exact incidence remains largely unknown [1]. PAIS involves the pulmonary trunk or proximal pulmonary arteries and exhibits clinical similarities to chronic pulmonary artery thromboembolism (CPTE), including dyspnea, chest pain, and hemosputum. Extrapulmonary metastases, in contrast to lung metastases, are less common, so that PAIS is rarely diagnosed with initial symptoms related to an extrapulmonary metastasis. Here, we describe a unique case of PAIS demonstrating an abnormal shadow on chest radiography during a health checkup in a patient without any cardiopulmonary symptoms.

## 2. Case Presentation

**Figure 1:**
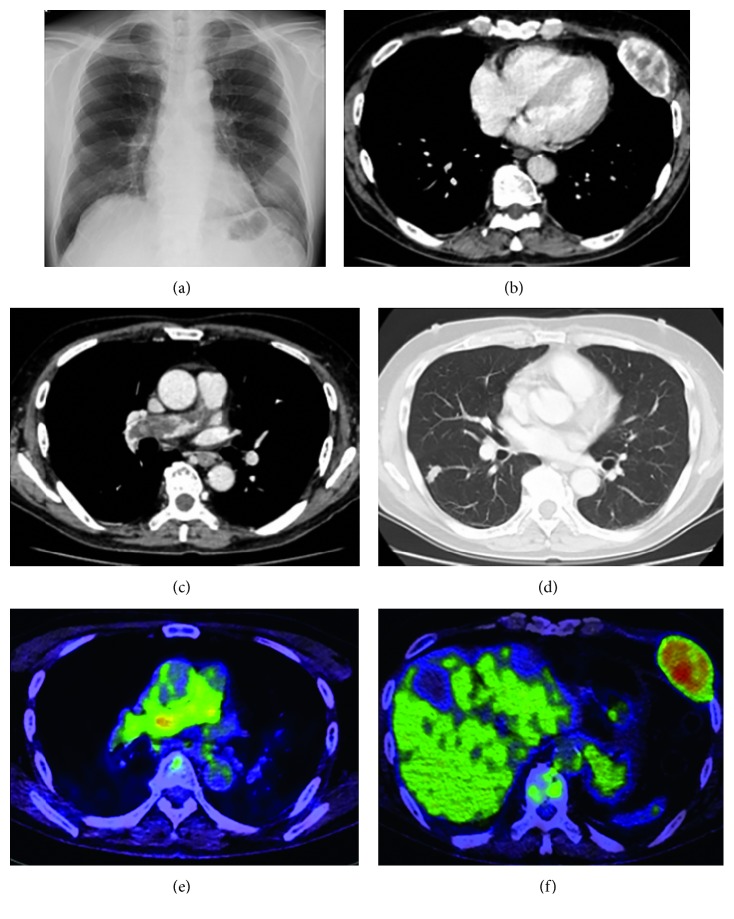
Radiological examinations at diagnosis. A chest radiograph showed a faint infiltrative shadow in the left lower lung field (a). Contrast medium-enhanced chest computed tomography showed a large tumor in the left anterior chest wall (b). An enhanced mass in the right pulmonary artery (c) and small nodules in the right lung (d) were also found. Fluorodeoxyglucose (FDG)-positron emission tomography revealed positive FDG uptake in the pulmonary artery mass (e) and in the chest wall tumor (f).

An 82-year-old man without any symptoms was referred to Hino Municipal Hospital, because of a faint infiltrative shadow observed in the left lower lung field on routine chest radiography (Figure 1(a)). The patient did not complain of chest wall swelling; however, the physical examination revealed a subcutaneous soft tumor on the left anterolateral chest wall without tenderness or a skin surface abnormality. The transcutaneous oxygen saturation of peripheral artery was 94% under the room air inhalation at the diagnosis. He had never smoked previously and was taking medication for hypertension and asymptomatic cerebral infarction. Contrast medium-enhanced chest computed tomography (CT) revealed an enhancing tumor (58 × 33 mm in size) on the left anterolateral chest wall with destructive changes in the 6th rib bone (Figure 1(b)), corresponding to the abnormal shadow on the chest radiograph. In addition, a massive filling defect of the right proximal pulmonary artery was detected with pulmonary nodules in the right lung (Figures 1(c) and 1(d)). Importantly, the “mass” in the pulmonary artery was, unlike in CPTE, weakly enhanced with contrast medium. Pulmonary nodules existed only on the ipsilateral side of the pulmonary artery mass and formed a mold-like shape in the peripheral pulmonary arteries. Further examination did not identify another site of suggestive malignancy, except for prostate cancer using body CT and head magnetic response imaging (MRI). A laboratory test did not show any significant abnormalities, including those of D-dimer and tumor markers. These findings implied a possible diagnosis of primary sarcoma of the pulmonary artery with metastases to the lungs and chest wall.

The patient was referred to Keio University Hospital for further examinations. Fluorodeoxyglucose (FDG)-positron emission tomography (PET) showed a positive uptake of FDG to the pulmonary artery tumor (standardized uptake value, SUVmax 5.32), the chest wall tumor (SUVmax 9.56), and lung nodules (highest SUVmax 3.50) (Figures 1(e) and 1(f)), strongly suggesting a primary malignant tumor of the pulmonary artery, but not the thromboembolism. Transcutaneous core needle biopsy was performed to obtain a tumor specimen from the chest wall. Microscopic examination revealed the atypical cells proliferating among the collagen fibers in hematoxylin-eosin staining. The results of immunostaining were as follows: pan-cytokeratin (−), CAM5.2 (−), epithelial membrane antigen (−), thyroid transcription factor-1 (−), calretinin (−), D2-40 (−), cluster of differentiation (CD)138 (+, focal), smooth muscle actin (+), HHF-35 (+), h-caldesmon (+/−), desmin (−), CD34 (−), CD31 (+/−), erythroblast transformation-specific related gene (+), S100 (−), human melanoma black 45 (−), and melan A (−). Approximately 30% of tumor cells were positive for Ki-67. While some markers for smooth muscle cells exhibited positivity, the cell morphology was inconsistent with that of leiomyosarcoma. A specific immunostaining pattern suggestive for the differentiated epithelial or mesothelial tumor was lacking. A histological diagnosis was finally determined to be unclassified sarcoma, which included a possible intimal sarcoma. The patient was diagnosed with PAIS on the basis of clinical, radiological, and pathological correlations.

**Figure 2:**
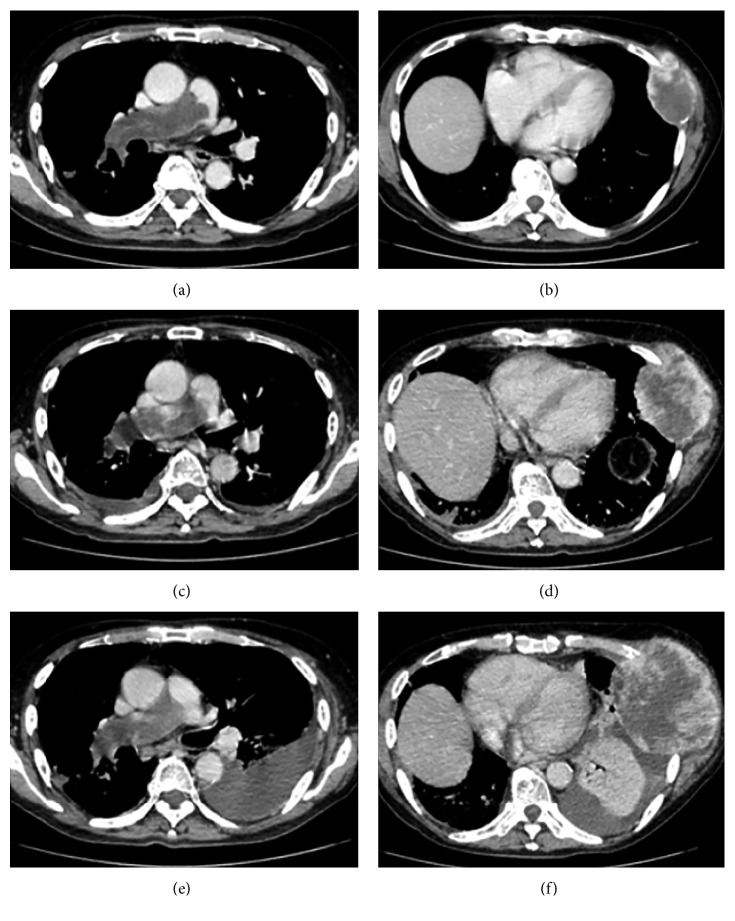
Chronological progression of the tumors on chest computed tomography. Computed tomography cross sections of the right pulmonary artery and chest wall tumor levels were shown at 4 (a, b), 8 (c, d), and 9 months (e, f) from the initial visit. The tumor expansively occupied the entire lumen of the right pulmonary artery, and the chest wall tumor drastically enlarged over time, with a massive left pulmonary effusion appearing at 9 months.

The patient was scheduled to receive supportive care in the outpatient clinic of Hino Municipal Hospital, because no chemotherapy regimen was established for elderly patients with PAIS. At that time, 2 months had passed from his initial visit, and he lived a further 10 months after the diagnosis. During the follow-up period, he developed progressive dyspnea on exertion and on rest. Chest CT showed expansive growth of the pulmonary artery tumor at 4, 8, and 9 months from the first visit to occupy the entire lumen, while the pulmonary trunk and left pulmonary artery were not involved (Figures 2(a), 2(c) and 2(e)). The lung nodules increased in size and number. The chest wall tumor drastically expanded in 9 months (131 × 113 mm in size) from the subcutaneous soft tissue to the thoracic cavity (Figures 2(b), 2(d) and 2(f)). The left lung received passive atelectasis with the chest wall tumor and accompanying pleural effusion. He died because of respiratory failure, and an autopsy was performed with consent from the patient's family.

**Figure 3:**
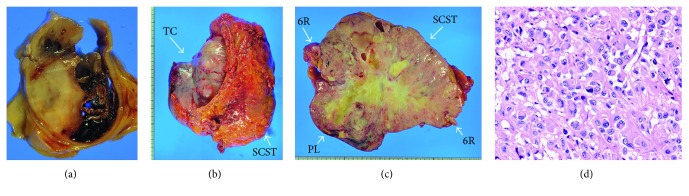
Pathological findings at autopsy. Macroscopic observation of the transverse cut surface of the right pulmonary artery showed the vascular lumen filled with the solid tumor and secondary thrombus (a). Gross appearance of the chest wall tumor that is occupying the full thickness from the soft tissue through the thoracic cavity (b). The cut surface of the chest wall tumor appeared as a white-colored solid mass with central necrosis (c). The left 6th rib bone showed the tumor with complete osteolysis. Microscopic observation in the pulmonary artery tumor showed that the tumor was predominantly composed of pleomorphic, multinucleated round tumor cells with an incohesive nature (d). TC, thoracic cavity; SCST, subcutaneous soft tissue; PL, pleura; 6R, 6th rib bone.

Macroscopic observation showed entire occlusion of the right pulmonary artery with a growing tumor and thrombus (Figure 3(a)). The tumor showed transmural expansion under microscopic observation in a limited portion of the right pulmonary artery wall. In addition, the tumor subsequently invaded the subintimal layer of the pulmonary trunk wall reaching the pulmonary valve. Multiple pulmonary metastases in each lobe of the right lung grew from microscopic levels to centimeter levels, while no metastasis existed in the left lung. A large tumor (170 × 160 mm in size) occupied the left anterolateral chest wall from the subcutaneous soft tissue through the thoracic cavity with involvement of the parietal pleura (Figure 3(b)). A large amount of hemorrhagic pleural effusion was present but was negative for malignancy on cytology. Since osteolysis of the 6th rib bone was apparent at the center of the chest wall tumor (Figure 3(c)), it was speculated that the chest wall tumor was arising from a bone metastasis. Despite a thorough whole-body examination, other sites of extrapulmonary metastasis were absent. The fatal respiratory failure was presumably attributed to the tumorous occlusion of the right pulmonary artery and the left lung compression. The microscopic findings were similar among the tumors in the pulmonary artery, lungs, and chest wall. The tumor was predominantly composed of pleomorphic, multinucleated round tumor cells with an incohesive nature and partly contained a hemangiopericytoma- or an angiosarcoma-like appearance, consisting of a pleomorphic sarcoma (Figure 3(d)). Immunostaining patterns were similar to those observed in the antemortem biopsy specimen of the chest wall.

## 3. Discussion

We described an asymptomatic case of PAIS demonstrating an abnormal shadow in the chest on a routine health checkup. This rare malignancy is often difficult to be diagnosed antemortem, since the clinical and radiological manifestations often mimic those in CPTE and mislead the diagnosis. A careful assessment is required to recognize the faint enhancement of the pulmonary artery mass on contrast medium-enhanced CT scans or gadolinium-enhanced MRI [2]. Recently, the utilization of FDG-PET was proposed as a superior modality to discriminate PAIS from thromboembolism by detecting high FDG uptake in the pulmonary artery mass [3, 4], whereas the cases were also reported to show negative FDG uptake in PAIS [5, 6]. In the present case, the coexistence of ipsilateral pulmonary nodules and the chest wall tumor provided a clue for the possible diagnosis of PAIS, and high FDG uptake in the pulmonary artery mass strongly supported the diagnosis. Another aspect contributing to diagnostic difficulty in PAIS is that the tumor specimens for pathological diagnosis are hard to obtain, except in surgical cases. Only a few studies have demonstrated the successful diagnosis of pulmonary artery sarcoma based on percutaneous endovascular catheter biopsy [7, 8]. In the present case, transcutaneous core needle biopsy of the chest wall was chosen for fast and safe diagnosis.

An initial symptom in PAIS is related predominantly to the pulmonary artery embolism, but merely to the extrapulmonary metastasis. We summarized three case series studies that described the initial manifestation in patients with PAIS [9–11]. A total of 30 PAIS cases were collected, in which the cardiopulmonary symptoms were described as an initial manifestation in 29 of 30 patients (in 1 case, initial symptoms were not determined), while no patient had extrapulmonary symptom. On the other hand, however, others have reported rare cases of PAIS, with patients demonstrating noncardiopulmonary symptoms as initial manifestations, such as cauda equina syndrome [12] and diarrhea [13]. Asymptomatic radiological abnormalities in routine health checkups are a unique presentation for PAIS and have not been previously reported. We speculate the given reason for being asymptomatic in the present case included an early diagnostic opportunity due to the abnormal shadow in the chest before accompanying severe occlusion of the pulmonary artery. Indeed, CT showed an intact enhancement of the right peripheral pulmonary arteries, and the transthoracic echocardiography did not show pulmonary artery hypertension, suggesting that blood flow in the right pulmonary artery was spared at diagnosis.

Because of the rarity and diagnostic difficulty in PAIS, the prevalence of metastases at intra- and extrapulmonary sites was not accurately determined. In the case series studies described above [9–11], about half of the cases (14 in 30 cases) had at least 1 metastasis at diagnosis. The most common site of metastasis was the lungs (13 in 14 cases), while extrapulmonary metastases were detected only in 6 cases. The sites of extrapulmonary metastases included the lymph nodes (3 cases), kidneys (3 cases), brain (2 cases), adrenal gland (1 case), skin (1 case), and bone (1 case), and multisite metastases were usual (4 in 6 cases). Only 1 case involved a single extrapulmonary metastasis of the kidney without pulmonary metastasis, and another case showed combined metastases of the lungs and skin. Metachronous metastases developed in all 6 cases, with newly appearing extrapulmonary metastases being detected between 4 and 8 months of the follow-up period [10]. In contrast to the previous studies, the present case is rare in terms of demonstrating a single extrapulmonary metastasis at the diagnosis throughout the 1 year of follow-up.

In summary, we described a unique case of asymptomatic PAIS demonstrating chest wall metastasis as an initial presentation. Physicians should be aware that PAIS is rarely diagnosed in asymptomatic patients.

## References

[1] Fletcher C., Bridge J., Hogendoorn P. (2015). *WHO Classification of Tumours of Soft Tissue and Bone*.

[2] Viana-Tejedor A., Mariño-Enríquez A., Sánchez-Recalde A. (2012). Intimal sarcoma of the pulmonary artery: diagnostic value of different imaging techniques. *Revista Española de Cardiología*.

[3] Ote E. L., Oriuchi N., Miyashita G. (2011). Pulmonary artery intimal sarcoma: the role of ^18^F-fluorodeoxyglucose positron emission tomography in monitoring response to treatment. *Japanese Journal of Radiology*.

[4] Tachihara M., Tanaka Y., Zen Y. (2017). The notable appearance of pulmonary artery intimal sarcoma on positron emission tomography (PET)/CT. *Internal Medicine*.

[5] Lee D. H., Jung T. E., Lee J. H. (2013). Pulmonary artery intimal sarcoma: poor ^18^F-fluorodeoxyglucose uptake in positron emission computed tomography. *Journal of Cardiothoracic Surgery*.

[6] Jiang S., Li J., Zeng Q. (2017). Pulmonary artery intimal sarcoma misdiagnosed as pulmonary embolism: a case report. *Oncology Letters*.

[7] Guirola J. A., Laborda A., De Gregorio M. A. (2017). Percutaneous intravascular biopsy using a bronchoscopy forceps diagnosis of a pulmonary artery intimal sarcoma. *Cardiovascular and Interventional Radiology*.

[8] Winchester P. A., Khilnani N. M., Trost D. W. (1996). Endovascular catheter biopsy of a pulmonary artery sarcoma. *American Journal of Roentgenology*.

[9] Burke A. P., Virmani R. (1993). Sarcomas of the great vessels. A clinicopathologic study. *Cancer*.

[10] Penel N., Taieb S., Ceugnart L. (2008). Report of eight recent cases of locally advanced primary pulmonary artery sarcomas: failure of Doxorubicin-based chemotherapy. *Journal of Thoracic Oncology*.

[11] Secondin S., Grazioli V., Valentino F. (2017). Multimodal approach of pulmonary artery intimal sarcoma: a single-institution experience. *Sarcoma*.

[12] Rashid A., Molloy S., Lehovsky J. (2008). Metastatic pulmonary intimal sarcoma presenting as cauda equina syndrome: first report of a case. *Spine*.

[13] Xu X., Zhang R., Hu H. (2015). Diarrhea as initial manifestation of pulmonary artery intimal sarcoma: a case report and literature review. *OncoTargets and Therapy*.

